# Endothelial Dysfunction in Atherosclerosis: Experimental Models and Therapeutics

**DOI:** 10.34133/bmr.0252

**Published:** 2025-10-08

**Authors:** Vadym Kopych, Avelino Dos Santos Da Costa, Kwideok Park

**Affiliations:** ^1^ Center for Biomaterials, Korea Institute of Science and Technology (KIST), Seoul 02792, Republic of Korea.; ^2^ Division of Bio-Medical Science and Technology, KIST School, University of Science and Technology (UST), Seoul 02792, Republic of Korea.

## Abstract

Atherosclerosis is a cardiovascular disease that involves complex and multifactorial processes that are instigated from endothelial dysfunctions. In this review, we address endothelial dysfunction in atherosclerosis and the mechanisms, where they are characterized by structural and functional alterations in endothelial cells (ECs), as caused by inflammation, oxidative stress, or disturbed shear stress. In particular interest, we provide a comprehensive overview of the experimental models (in vitro and in vivo) used to investigate endothelial dysfunction, specifically the role of ECs in atherosclerosis. Finally, current therapeutics, for example, pharmacological interventions, cell and gene therapies, and nanomedicine, are introduced, along with emerging technologies for advanced treatment of atherosclerosis. This review will help readers better understand current scientific findings, experimental models, and technologies in an effort to decipher the mechanisms of atherosclerosis and to advance therapeutic interventions.

## Introduction

### Atherosclerosis: A chronic vascular disease

**Fig. 1.**
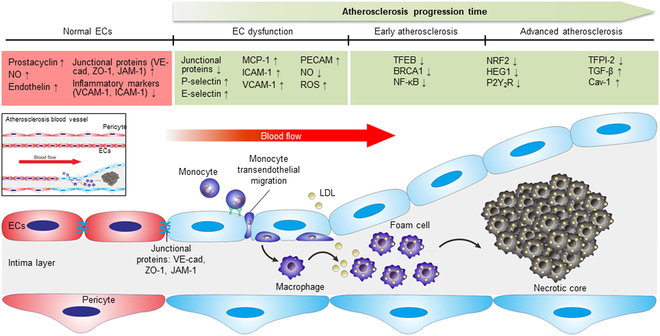
Schematic illustration depicting the time-dependent progression of atherosclerosis disease. In healthy ECs, they express higher prostacyclin, NO, endothelin, and junctional proteins, such as VE-cad, ZO-1, and JAM-1. Inflammatory markers, such as VCAM-1 and ICAM-1, also remain lower under normal EC function. Conversely, atherosclerosis begins from the endothelial dysfunction as marked by the up-regulation of inflammatory markers, such as P-selectin, E-selectin, ICAM-1, and VCAM-1, which then lead to the down-regulation of junctional proteins. Consequently, this causes monocyte adhesion and transendothelial migration into the intima layer where these monocytes are then differentiated into macrophages. Moreover, as the LDL in the bloodstream is infiltrated and absorbed by these macrophages, they further differentiate into foam cells, known as the early sign of atherosclerosis. EC dysfunction also induces the up-regulation of MCP-1, ROS, and PECAM-1, which escalate the development of early atherosclerosis. In early and advanced atherosclerosis stage, major markers down-regulated are TFEB, BRCA1, NF-κB, NRF2, HEG1, P2Y_2_R, and TFPI-2. Conversely, those up-regulated are TGF-β and Cav-1. Formation of necrotic core is the sign of advanced stage of atherosclerosis, which is due to the persistent accumulation and growth of foam cells. NO, nitric oxide; VE-cad, vascular endothelial-cadherin; ZO-1, zonula occludens-1; JAM-1, junctional adhesion molecule 1; VCAM-1, vascular cell adhesion molecule 1; ICAM-1, intercellular adhesion molecule-1; LDL, low-density lipoprotein; MCP-1, monocyte chemoattractant protein-1; ROS, reactive oxygen species; PECAM-1, platelet endothelial cell adhesion molecule-1; TFEB, transcription factor EB; BRCA1, breast cancer gene 1; NF-κB, nuclear factor κB; NRF2, nuclear factor erythroid 2-related factor 2; HEG1, heart development protein with EGF-like domains 1; P2Y_2_R, P2Y_2_ receptor; TFPI-2, tissue factor pathway inhibitor-2; TGF-β, transforming growth factor β; Cav-1, caveolin-1. Inset is the narrower of the blood vessel in vascular atherosclerosis development.

Atherosclerosis, a chronic inflammatory disease of the arterial wall, is fundamentally driven by endothelial dysfunction—a pathological turnover of the innermost layer of blood vessels [1,2]. This dysfunction is followed by a cascade of events leading to the development of atherosclerotic plaques [3]. As depicted in Fig. 1, a key initiating event involves the compromised integrity of ECs. Such ECs would exhibit altered permeability, allowing the subendothelial retention and modification of circulating lipoproteins, particularly low-density lipoprotein (LDL). This accumulation triggers an inflammatory response [4]. Circulating leukocytes and T lymphocytes are then recruited to the inflamed endothelium, mediated by the up-regulation of specific adhesion molecules on the surface of activated ECs [5]. These monocytes subsequently infiltrate the arterial wall, differentiate into macrophages, and then take up modified LDL, and eventually being transformed into lipid-laden foam cells [6,7]. The progressive accumulation of these foam cells and other immune cells, alongside extracellular matrix (ECM) deposition, shapes up the characteristic atherosclerotic plaque (Fig. 1). The pathological development of cardiovascular disease (CVD) results in clinically severe outcomes, such as peripheral artery disease, myocardial infarction, and stroke. Atherosclerosis is a leading cause of CVD. According to the World Health Organization (WHO), cardiovascular disorders are the top cause of death globally, and thus, the impact to the healthcare systems is vast. Despite substantial advancements in cardiovascular research, encompassing new experimental models and emerging therapeutic approaches, the complexity of endothelial dysfunction in atherosclerosis still requires in-depth science and interdisciplinary medical research. While several review literatures have explored individual aspects of endothelial dysfunction or specific therapeutic modalities, this review offers a comprehensive overview that includes both traditional and novel experimental models in a particular interest, along with the scientific background of endothelial dysfunction in atherosclerosis and introduction of various therapeutic development.

### Endothelial cells in atherosclerosis

The monolayer of cells that covers the inner surface of blood vessels is endothelial cells (ECs). They play a vital role in acting as an interface between the blood and the vascular wall. Through the regulation of numerous physiological processes, such as vascular tone, blood fluidity, and immune responses, they are essential in the maintenance of vascular homeostasis [8]. In order to carry out its regulatory mission, the endothelium secretes a number of biomolecules, including nitric oxide (NO), prostacyclin, and endothelin [9]. These substances are crucial for regulating leukocyte adhesion, blocking platelet aggregation, and controlling vasodilation and constriction. In this regard, endothelial dysfunction is a major detrimental contributor of the initiation and progression of vascular diseases in the context of atherosclerosis. A shift from a protective, anti-inflammatory, and anticoagulant state to a pro-inflammatory and pro-thrombotic phenotype is an indicator of endothelial dysfunction [10]. Reduced NO bioavailability, elevated reactive oxygen species (ROS) generation, and overexpression of adhesion molecules, i.e., VCAM-1 and ICAM-1, are the hallmarks of such changes [11,12]. These alterations would cause excessive inflammation and facilitate leukocyte adherence and transendothelial migration and oxidized LDL (Ox-LDL) [13], all of which contribute to the foam cell development and plaque formation with time. Moreover, endothelial dysfunction exacerbates fat deposition within the artery wall by increasing the endothelium’s permeability to lipoproteins and other circulating molecules [14]. It also activates vascular smooth muscle cells (VSMCs), where they produce excessive ECM [15] and thus take part in the plaque growth and maturation.

## Causes and Mechanisms of Endothelial Dysfunction

### Structural and functional alterations in ECs

Endothelial dysfunction in atherosclerosis is a multifactorial process driven by complex interplay of various pathological stimuli. While often introduced separately, these causative factors—chronic inflammation, oxidative stress, and disturbed blood flow—are highly interconnected and mutually contributing to vascular damage and disease progression. Understanding these intricate overlaps is crucial for developing not only physiologically relevant models but also effective therapeutic strategies. Morphologically, ECs undergo enlargement and increased permeability due to their structural alterations [9]. Cytoskeletal changes disrupt cell–cell connections, widening intercellular gaps and compromising the endothelial barrier, allowing easier infiltration of lipoproteins and monocytes into the subendothelial space [16]. ECs also lose their ability to maintain vascular homeostasis due to reduced NO production and bioavailability [17]. NO, produced by endothelial nitric oxide synthase (eNOS), is vital for vasodilation, inhibiting platelet aggregation, and preventing leukocyte adhesion [18,19]. Atherosclerosis is marked by reduced NO availability [20,21], often due to its interaction with superoxide anions forming peroxynitrite—a harmful molecule that heightens oxidative stress and damages ECs [22–24]. Normally, ECs express anticoagulant factors like endothelial protein C receptor and thrombomodulin [25]. In dysfunction, a pro-thrombotic phenotype would emerge with increased thrombogenic molecules and reduced anticoagulants [26], raising the risk of thrombosis on plaques, potentially causing myocardial infarction or stroke.

### Inflammation

Endothelial dysfunction is also caused by inflammation. ECs release pro-inflammatory cytokines and chemokines in response to endothelial injury or disruption. These include interleukin-6 (IL-6), tumor necrosis factor-α (TNF-α), and monocyte chemotactic protein-1 (MCP-1) [27,28]. They would attract immune cells, particularly monocytes, promoting chronic arterial wall inflammation and plaque progression [29]. Inflammatory cytokines not only promote plaque growth but also weaken the plaques, increasing their likelihood of rupture and leading to major cardiovascular symptoms [30,31]. A biomarker, such as high-sensitivity C-reactive protein (hs-CRP), indicates systemic inflammation and cardiovascular risk [32]. Ox-LDL is crucial for initiating and sustaining inflammation in plaque formation [33,34], activating toll-like receptors (TLRs) on ECs and macrophages, and encouraging foam cell formation [35]. This establishes a cycle of lipid accumulation and immune activation, eventually accelerating plaque development. Adaptive immunity is also involved—T helper cells infiltrate plaques and release interferon-γ (IFN-γ), amplifying inflammation [36], while regulatory T cells (Tregs) offer a counteracting anti-inflammatory effect [37].

### Oxidative stress

Chronic inflammation, a central player in endothelial dysfunction, facilitates oxidative stress and is, in turn, exacerbated by oxidative stress. For instance, inflammatory cytokines, such as TNF-α and IL-6, up-regulate NADPH (reduced form of nicotinamide adenine dinucleotide phosphate) oxidases in ECs, leading to increased production of ROS. Conversely, elevated ROS levels can activate pro-inflammatory signaling pathways [e.g., nuclear factor κB (NF-κB)], creating a vicious cycle that perpetuates endothelial damage. Oxidative stress, caused by an imbalance between ROS and antioxidants, exacerbates endothelial dysfunction and atherosclerosis [38]. Major ROS sources in ECs include uncoupled eNOS, NADPH oxidase, xanthine oxidase, and the mitochondrial electron transport chain [39–41]. ROS reduces NO bioavailability and promotes a pro-thrombotic state with elevated vascular tone and impaired vasodilation. Peroxynitrite, formed from ROS and NO, further damages ECs [42]. While antioxidant therapies showed a promise, oxidative stress contributes to vascular dysfunction via multiple mechanisms. ROS activates redox-sensitive transcription factors like activator protein-1 (AP-1) and NF-κB [43,44], which up-regulate pro-inflammatory cytokines IL-1β, TNF-α, and IL-6 [45–47]. ROS also influences VSMCs, promoting their migration and proliferation—key steps in plaque and fibrous cap formation [48]. In later stages, ROS would induce VSMC apoptosis, weakening plaques [49], and stimulate vascular calcification via osteogenic signaling, leading to arterial stiffness [50].

### Disturbed flow

Hemodynamic forces, particularly disturbed blood flow, play a critical role in initiating endothelial dysfunction and atherosclerosis [51,52]. Plaques often form in arterial bifurcations and curvatures exposed to oscillatory flow, unlike the areas with laminar flow [53,54]. Low and disturbed shear stress negatively influence EC gene expression and behavior [55], leading to a pro-inflammatory, pro-oxidative phenotype with up-regulated expression of adhesion molecule and cytokine [56,57]. Shear stress disruption also affects EC alignment and function [58]. Disturbed flow activates mechanotransduction pathways, such as NF-κB and other inflammatory transcription factors [59]. Mechanosensitive structures—integrins, ion channels, and glycocalyx—translate mechanical stimuli into biochemical signals [60]. One key pathway involves Krüppel-like factor 2 (KLF2), which triggers anti-inflammatory responses under laminar flow but is suppressed under disturbed flow [61]. Disturbed flow suppresses KLF2, promoting a pro-inflammatory and pro-oxidative state in ECs, whereas laminar flow increases KLF2 expression, encouraging anti-inflammatory and antioxidative responses [62]. The mitogen-activated protein kinases (MAPKs) [52], including c-Jun N-terminal kinases (JNKs) [63] and extracellular signal-regulated kinases (ERK1/2) [64], are also involved in another crucial mechanism of mechanotransduction. These kinases are activated in response to disturbed flow, up-regulating the expression of adhesion molecules VCAM-1 and ICAM-1, consequently facilitating leukocyte adhesion on ECs and infiltrations. MAPKs enhance vascular inflammation by mediating the synthesis of pro-inflammatory cytokines. This signaling promotes EC activation, immune cell recruitment, and plaque development.

## Experimental Models for Studying ECs in Atherosclerosis

Atherosclerosis remains a leading cause of mortality worldwide, posing a remarkable public health burden [65,66]. Despite the advances in medical management, there is still a pressing need for a deeper understanding of the underlying mechanisms and the development of more effective and robust therapeutic interventions as well. In this section, we extensively introduce the experimental models of atherosclerosis in vitro and in vivo, based on a series of pathological stages, such as endothelial dysfunction, early atherosclerosis stage, including monocyte recruitment, lipid accumulation, and foam cell formation, and advanced atherosclerosis stage with plaque formation.

**Fig. 2.**
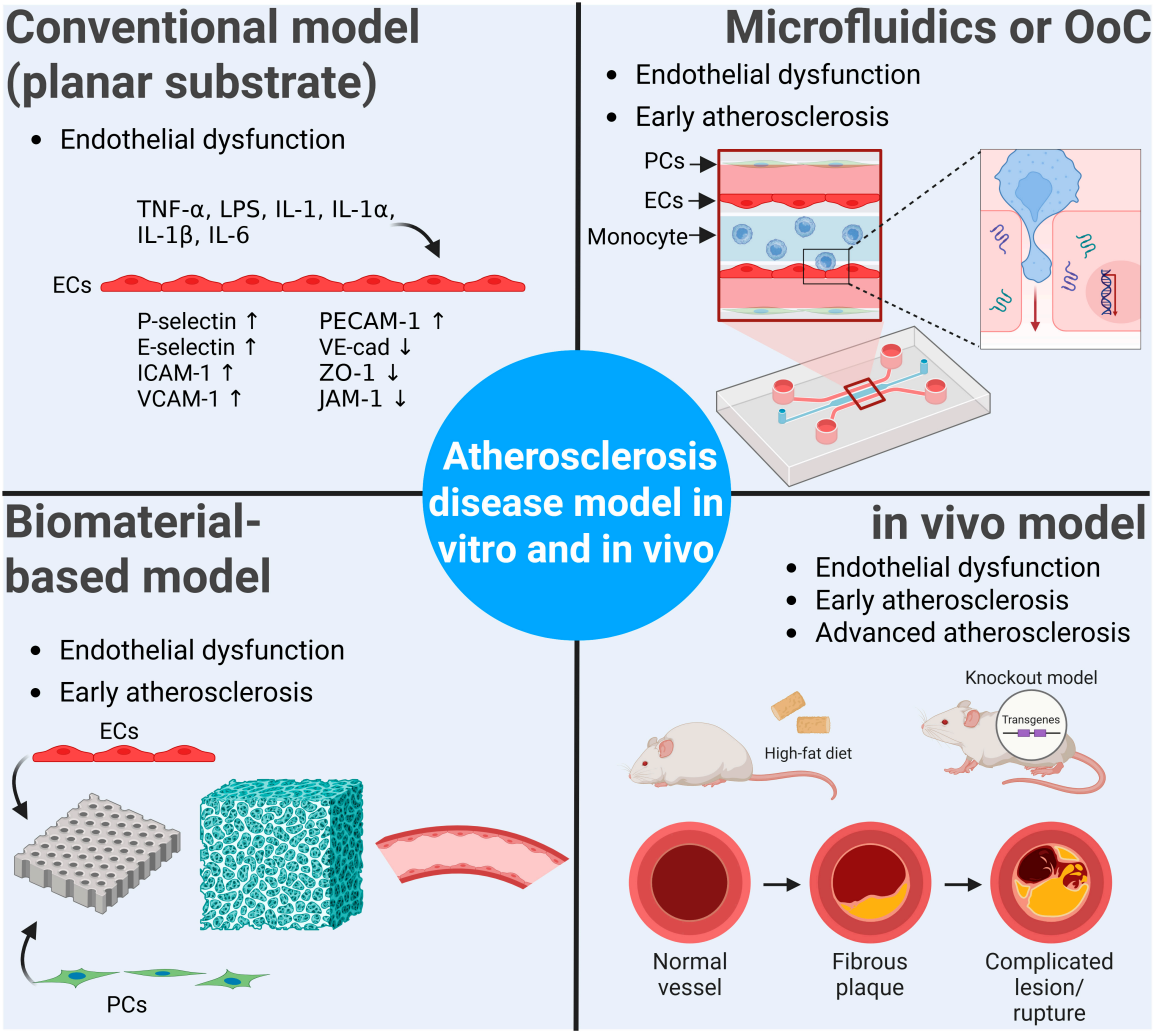
Overview of the current experimental models for studying EC dysfunction in atherosclerosis. A conventional model that uses a planar substrate, i.e., tissue culture plastic, is common and standardized in growing ECs in monolayer. This model is easy to access and very useful in testing the impact of cytokines, such as TNF-α, LPS, IL-1, IL-1α, IL-1β, and IL-6 on EC dysfunction. Inflammatory markers (P-selectin, E-selectin, ICAM-1, VCAM-1, and PECAM-1) and junctional proteins (VE-cad, ZO-1, and JAM-1) are easily determined via conventional model. Meanwhile, microfluidics or OoC has emerged to better replicate endothelial dysfunction and early atherosclerosis formation. This model can handle different types of cells and examine their interactions in a coculture system. Biomaterial-based models have also been developed to better recapitulate healthy endothelium and EC dysfunction as well. This model harnesses various types of synthetic or natural polymers while integrating vascular cells with biomaterials in various design for replicating endothelial dysfunction and early atherosclerosis stage. Lastly, the animal model is a traditional and the most physiologically relevant model that can recapitulate all stages of atherosclerosis development, including endothelial dysfunction and early and late stage of atherosclerosis. The image is produced using BioRender.

Experimental models would provide invaluable tools for dissecting the molecular pathways governing the endothelial dysfunction and for evaluating the efficacy of potential therapeutic interventions. A summary of the recent advances of experimental models that are focused on the roles of ECs and pathologies in the study of atherosclerosis appears in Fig. 2.

### Conventional cell culture models

Tissue culture plate (TCP) has served as a simple and widely used in vitro model to study atherosclerosis, particularly focusing on EC dysfunction and early atherosclerosis development (Table 1). A common approach involves stimulating ECs with inflammatory cytokines, such as TNF-α, IL-1 variants, and IL-6 [67–73], which up-regulate key inflammatory markers like P-selectin, E-selectin, VCAM-1, ICAM-1, and PECAM-1 [74–80], triggering monocyte rolling and adhesion to the ECs in endothelial dysfunction [81]. Alternatively, EC dysfunction can be induced by oxidative stress via hydrogen peroxide (H₂O₂) treatment [82]. Zhang et. al. presented that overexpression of ACE2 and Ang-(1–7) inhibits inflammation by down-regulating VCAM-1 and E-selectin, thereby protecting against atherosclerosis [83]. In another study, KLF4 also plays a key anti-inflammatory role by suppressing VCAM-1 and promoting eNOS expression [84,85]. Moreover, magnesium deficiency in ECs can activate NF-κB, contributing to EC dysfunction [86]. During the early stage of atherosclerosis, inflamed ECs play a pivotal role in mediating the transendothelial migration of monocytes via loose cell–cell junctions or endocytosis [87,88], guided by chemoattractants and adhesion molecules [89]. Migrated monocytes differentiate into macrophages, which can be simulated by activating TCP-cultured monocytes with IL-32, lipopolysaccharide (LPS), or phorbol 12-myristate 13-acetate (PMA) [90–92]. Concurrently, dysfunctional ECs enhance LDL transfer to macrophages through caveolin-1-mediated endocytosis [93–95]. LDL is then oxidized by the ROS as assisted by the enzymes, such as NADPH oxidase (NOX), lipoxygenases (LOX), xanthine oxidase (XO), myeloperoxidase (MPO), cytochrome P450 (CYP)/cytochrome P450 reductase (POR), glucose oxidase (GOx), and uncoupled eNOS [96,97]. Such Ox-LDL eventually causes the endothelial dysfunction via the production of ROS and up-regulated inflammatory markers, i.e., VCAM-1, ICAM-1, E-selectin, and MCP-1 [98,99]. Despite its limited physiological relevance, TCP still remains a valuable model for investigating EC dysfunction and the early stages of atherosclerosis development (Table 2).

**Table 1.** Summary of in vitro and in vivo atherosclerosis models and key findings

Endothelial function and dysfunction		
TCP or planar substrate	Key findings	Refs.
Bovine aortic ECs on TCP	- Endothelial dysfunction is induced by TNF-α treatment, where it mediated the down-regulation of eNOS promoter, which then resulted in the down-regulated NO production, a precursor of endothelial dysfunction. - TNF-α treatment down-regulates NO production, which is the early sign of endothelial dysfunction.	[71]
HUVECs on TCP	- Endothelial dysfunction is induced by TNF-α treatment. TNF-α causes YAP dephosphorylation, which then up-regulates VCAM-1 and subsequently increases the monocyte adhesion. - TNF-α up-regulates VCAM-1 and subsequently increases the adhesion of monocyte via the activation of YAP-TAZ signaling.	[73]
Coculture of EA.hy296 human vascular cells and THP-1 on TCP	IL-1β-treated cells up-regulate ICAM-1 and then, subsequently, elevate the adhesion of THP-1 on human vascular cells.	[72]
HUVECs on TCP	Endothelial dysfunction is induced by the treatment of H_2_O_2_, which up-regulates oxidative stress.	[82]
HUVECs on TCP	Overexpression of ACE2 and Ang-(1–7) protects endothelial function by inhibiting the inflammatory response, thus suppressing atherosclerotic progress.	[83]
HUVECs on TCP	KLF4 inhibits the expression of inflammatory mediators, such as tissue factor and VCAM-1, and enhances the expression of eNOS and thrombomodulin.	[85]
HUVECs on 2% gelatin substrate	Low Mg induces endothelial dysfunction and endothelial inflammatory cytokines, thus boosting atherosclerosis.	[86]
**Microfluidics**		
HUVECs	Increased ROS level and contraction of ECs in the microfluidics under lower shear stress	[107]
Human aortic ECs (HAECs)	Disturbed shear stress causes the disruption of β-catenin in HAECs	[112]
Coculture of ECs and SMC	Vascular inflammation using IL-1β and TNF-α increased the migration of SMCs	[102]
Coculture of human retinal microvascular ECs and retinal pericyte	Increased inflammatory cytokines, such as IL-6, IL-8, G-CSF, and Fractalkine, when exposed to TNF-α	[109]
HUVECs	Up-regulated proinflammatory markers, such as ICAM-1 and VCAM-1, when treated with TNF-α	[110]
HAECs	Disturbed shear stress causes up-regulated ICAM-1 and enhanced permeability	[113]
Primary bovine aortic ECs	Fibronectin-coated hydrogel failed to form a robust VE-cad in bovine aortic ECs compared to collagen-coated one.	[114]
**Biomaterial-based**		
Coculture of HUVEC and MSC on microperforated PCL	This study finds the effect of ECs in up-regulating α-SMA in MSCs under the coculture system compared to monoculture of MSC. In atherosclerosis, α-SMA-positive MSC-derived cells play roles in plaque stability.	[121]
Coculture of HUVEC and MSC on dual microstructured PCL film	Interaction of HUVEC and MSC through the holes of the substrate resulted in relatively higher level of NO secretion.	[122]
Coculture of HUVEC and ADSC on SiO_2_ membrane	A successful coculture of HUVEC and ADSC on the SiO_2_ membrane.	[123]
Coculture of HUVEC and hMSC on PLCL membrane	A successful coculture of HUVEC and hMSC on elastic, porous ultrathin membrane (EPUM) using PLCL, disclosing the functionality of ECs, as characterized by tight endothelial barrier.	[124]
**Animal**		
EC-specific TFEB transgenic mice	Overexpression of EC-specific TFEB in ECs decreases the inflammatory marker VCAM-1	[138]
TNF^−/−^ mice	TNF-α induces in vivo endothelial dysfunction	[141]
Lepr^db^ and db^TNF−^/ db^TNF−^ mouse	TNF-α-activated NAD(P)H oxidase and the production of ROS lead to the endothelial dysfunction in type 2 diabetes.	[142]
EC-PRAS40-KO mice	PRAS40 suppresses the development of atherosclerosis. EC-PRAS40-KO mice up-regulate the pro-inflammatory signaling, as assessed by cell adhesion molecules VCAM-1 and ICAM-1.	[105]
ApoE^−/−^ eNOS-tg	Accelerated atherosclerosis by eNOS overexpression in apoE-KO mice.	[140]
ApoE^−/−^ and C57BL/6J mice	When treated with acetylcholine, Ca^2+^ ionophore and diethylammonium (*Z*)-1-(*N*,*N*-diethylamino)diazen-1-ium-1,2-diolate (DEA-NONOate) caused the increased production of O_2_^−^, which reduced NO activity.	[143]
Pecam1^−/−^ ApoE^−/−^ mice	PECAM-1 deficiency leads to the lower atherosclerosis in the region of disturbed flow. PECAM-1 controls NF-κB activation.	[139]
**Early stage of atherosclerosis**		
TCP, Transwell	Key findings	Refs.
HUVECs on TCP	Endothelial TFEB protects against atherosclerosis by inhibiting ROS and inflammation in ECs.	[138]
Triple culture of ECs, arterial SMCs, and human peripheral blood monocyte on transwell	Monocytes were differentiated into macrophages after transendothelial migration. Additionally, once treated with LDL, foam cell formation was also observed.	[95]
HUVECs on TCP	Ox-LDL treatment up-regulated phosphorylation of caveolin-1.	[94]
**Microfluidics**		
Coculture of HUVEC and THP-1	Shear stress up-regulated ICAM-1 and down-regulated IL-8 secretion from THP-1 cell. Shear stress also attenuated the binding of THP-1 on ECs.	[108]
Triple culture of vascular SMCs, HUVECs, and monocyte	Triple culture of vascular SMCs, ECs, and monocyte enabled the formation of more foam cells when LDL was treated, along with the mechanical stretching in the device.	[116]
Triple culture of vascular SMCs, ECs, and monocyte	Cocultured vascular SMCs, ECs, and monocyte enabled foam cell formation upon the treatment of Ox-LDL.	[117]
Triple culture of human brain derived pericytes, HUVEC, and THP-1	The number of THP-1 colocalized with pericytes (PCs) increases when treated with TNF-α, suggesting higher transendothelial migration rate.	[118]
Coculture of HAECs and peripheral blood mononuclear cells (PBMCs)	Endothelial dysfunction is induced by TNF-α treatment and exposure to disturbed shear stress. The number of PBMCs undergoing transendothelial migration increases under disturbed shear stress conditions.	[119]
Triple culture of HAVSMC, vascular SMCs, and THP-1	Endothelial dysfunction, as assessed by the up-regulated pro-inflammatory cytokines (MCP-1, IL-6, IL-8, IL1β, TNF-α, IFN-γ) and foam cell formation, is induced via disturbed shear stress.	[120]
**Biomaterial-based**		
Monoculture of HUVEC on nano- and microporous membrane	Leukocyte transmigration through the monolayer of ECs is observed with the addition of chemoattractant (*N*-formylmethionyl-leucyl-phenylalanine, fMLP) to the bottom chamber.	[125]
Coculture of HUVEC and human placental PCs on porous PEGDA hydrogel	ECs are responsive to TNF-α, and subsequently, once seeded with leukocytes, these leukocytes undergo transendothelial migration.	[126]
Coculture of PBMCs and HUVECs on collagen matrix	Upon TNF-α-induced endothelial dysfunction, there is the transendothelial migration of monocyte (PBMCs) into the collagen layer. PBMCs are then differentiated into foam cells without the addition of lipids.	[87]
Coculture of HUVECs and HCASMCs via in-bath coaxial printing	Endothelial dysfunction is induced via TNF-α, and subsequent seeding of THP-1 resulted in the transendothelial migration and differentiation into macrophage. Once treated with LDL, foam cell formation was confirmed by the accumulation of lipid uptake.	[127]
Triple culture of HUVEC, hMSC, and THP-1 on *p*-PVA hydrogel that embedded with ECM	ECs are responsive to TNF-α as noted by the up-regulation of ICAM-1 and VCAM-1 and loosening of VE-cad. Once seeded with monocytes, the monocytes undergo transendothelial migration via endothelial monolayer and subsequently developed foam cells when treated with Ox-LDL.	[128]
**Advanced stage of atherosclerosis**		
Animal	Key findings	Refs.
EC-specific ApoE^−/−^ ad-BRCA1 mice	ad-BRCA1 (BRCA1 adenovirus) mice showed the reduction of atherosclerosis lesion formation, due to less infiltration of macrophage into the aortic roots.	[144]
NEMO^EC-iKO^/ApoE^−/−^ mice	The ablation of NEMO/IKKγ reduced the plaque formation in ApoE^−/−^ mice.	[145]
EC-specific Nrf2 activation in ApoE^−/−^ mice or *Cdh5^Cre^Cas9^floxed-^* ApoE^−/−^ mice	EC-specific activation of Nrf2 inhibits atherosclerosis, while EC-specific knockout of Nrf2 results in the progression of atherosclerotic plaque in aortas and aortic roots.	[146]
Endothelial TGF-β receptors KO mice	Inhibition of endothelial TGF-β signaling in hyperlipidemic mice reduces vessel wall inflammation and vascular permeability.	[147]
HEG1^iECKO^ mice	HEG1 is a novel atheroprotective marker, as evidenced by the worsening of the atherosclerosis plaque development in HEG1^iECKO^ mice model.	[148]
Cav-1*^TG^* ApoE^−/−^ mice	Overexpression of Cav-1 in ECs decreased cell proliferation and migration, which then influenced the progression of atherosclerosis in mice.	[149]
EC-specific P2Y_2_R-deficient ApoE^−/−^ mice	EC-specific P2Y_2_R knockout reduces atherosclerotic burden and promotes plaque stability in ApoE^−/−^ mice.	[134]
TFPI-2^fl/+^/Tek-Cre mice	Monoallelic deletion of TFPI-2 gene in vascular ECs leads to significant down-regulation of TFPI-2. TFPI-2 deficiency may accelerate the initiation of atherosclerotic lesion in mice.	[135]

**Table 2.** In vitro and in vivo atherosclerosis models: Pros and cons

Type of models	Pros	Cons
TCP or planar substrate	Simple; reproducible; cost-effective; relatively high-throughput; observation of endothelial dysfunction	Poor physiological relevance; lack of shear flow; limited multicellular complexity; limited reproduction of early atherosclerosis; unaffordable of endothelial permeability measurement
Microfluidics	Mimicking of blood shear flow; 3D microenvironment for multicellular culture; reproduction of early atherosclerosis development; affordable of endothelial permeability study	Technically complex design; not easily reproducible; not scalable; difficulty of sample collection
Biomaterial-based	3D environment for multicellular culture; customizable materials; reproduction of early atherosclerosis development; affordable of endothelial permeability study	Limited physiological relevance; mostly static culture; difficulty of mimicking advanced atherosclerosis stage
Animal	High physiological relevance; long-term observation; reproduction of advanced atherosclerosis stage; target gene editing; affordable of endothelial permeability study	Batch-to-batch variations; time-consuming; high cost; ethical concerns

### Microfluidics and organ-on-a-chip models

This section outlines current state-of-the-art of microfluidics and organ-on-a-chip (OoC) technology to deepen our understanding about the roles of ECs in atherosclerosis. Microfluidics is focused on the manipulation of fluidics in a chip [100,101], whereas OoC is primarily interested in recapitulating specific tissues or organs in a chip system [102,103]. In some cases, these are integrated to replicate in vitro atherosclerosis model [104]. In general, microfluidics and OoC models are considered to be a physiologically relevant one compared to conventional TCP, due mainly to their ability to enable integration of multiple biomimetic components on the spot, such as various cell types [105], 3-dimensional (3D) microenvironment, and external signaling cues (Table 2). Both systems can mimic endothelial dysfunction and early stage of atherosclerosis development. An endothelial dysfunction stage using either microfluidics or OoC can be realized by applying inflammatory cytokines, such as TNF-α [106] and/or shear stress, especially disturbed shear stress [107] or a combination of both [108]. For example, in a microfluidic model of coculturing human retinal ECs and pericytes (PCs), exposure to TNF-α leads to the up-regulation of several inflammatory cytokines, including IL-6, IL-8, granulocyte colony-stimulating factor (G-CSF), and Fractalkine [109]. Another study also showed that TNF-α-treated human umbilical vein endothelial cells (HUVECs) in microfluidics were up-regulated with the level of ICAM-1 and VCAM-1 expression [110].

Meanwhile, disturbed shear stress has also been employed in microfluidics and OoC in order to induce endothelial dysfunction where atherosclerosis tends to be developed at the arterial branches and curved regions [111]. When ECs are exposed to disturbed shear stress, ROS level increases, the hallmark of the early atherosclerosis [107]. ROS is widely recognized as a key factor contributing to triggering endothelial dysfunction, which then accelerates the progression of atherosclerosis [97]. Additionally, disturbed shear stress also disrupts β-catenin in ECs, a positive regulator of eNOS activity [112]. Furthermore, endothelial dysfunction of human aortic ECs (HAECs) under disturbed shear stress discloses higher ICAM-1 expression and increased permeability as marked by the lower transendothelial electrical resistance (TEER) [108,113]. Another mechanism of endothelial dysfunction is related to the microenvironment. A study using primary bovine aortic ECs cultured on either fibronectin (Fn) or collagen highlighted that the cells on Fn-coated microchannels exhibited higher permeability than those on collagen-coated channels, due mainly to the difference in vascular endothelial-cadherin (VE-cad) morphology [114]. This study suggested that endothelial dysfunction increases Fn expression, leading to VE-cad loosening and elevated permeability accordingly.

In recapitulating early atherosclerosis, microfluidics or OoC model is also an effective tool in elucidating the mechanisms behind monocyte transendothelial migration [102,115]. Researchers have gained insights about the role of ECs in the process of the extravasation of human monocyte, THP-1, into the intima layer. Microfluidic platform is utilized to assess the formation of foam cells in early atherosclerosis stage. Coculture of HUVECs and VSMCs in a stretchable microfluidic, along with the LDL treatment, enabled the formation of foam cells, as assessed by the up-regulation of Oil Red O staining [116]. Although the role of ECs was unclear, foam cells could also be developed by introducing Ox-LDL in the microfluidic that contained multiple cells, monocytes, ECs, and VSMCs [117]. Transendothelial migration of monocytes across the EC monolayer is readily induced via TNF-α. For example, a coculture system involving HUVECs and human brain-derived PCs demonstrated that the majority of the monocytes underwent transendothelial migration [118]. The impact of shear stress can also be evaluated using microfluidics or OoC. When the microfluidics system held HAECs and peripheral blood mononuclear cells (PBMCs) under disturbed shear stress, the number of cells under the transendothelial migration significantly increased, suggesting that disturbed shear stress could promote the migration of monocytes [119].

Another key benefit of using microfluidics or OoC platform is the ability of this platform to mimic the cascades of the multicellular events, from the endothelial dysfunction to the formation of foam cells. An example is the triple culture of HUVECs (EAhy926), human aortic vascular smooth muscle cells (HAVSMCs), and THP-1 in a microfluidics while providing the disturbed shear stress [120]. As a result, endothelial dysfunction was confirmed by the up-regulation of various proinflammatory cytokines, such as MCP-1, IL-6, IL-8, IL1β, TNF-α, and IFN-γ. After then, foam cells were also observed, with the up-regulation of Oil Red O and Bodipy staining. In particular interest, these multicellular events required only pressure and shear stress.

Overall, microfluidics and OoC models offer distinct advantages for studying ECs in the context of physiological relevance, distinguished from the traditional in vitro methods. These systems enable the application of shear stress, particularly disturbed shear stress, which closely mimics the physiological conditions where atherosclerosis predominantly develops at arterial branches and curves. By replicating such physiological environment, these technologies have the great potential to deepen our understanding of atherosclerosis and should accelerate the development of advanced treatments.

### Biomaterial-based models

Apart from the aforementioned in vitro models, various biomaterials have been employed to handle ECs and prepare a biomimetic atherosclerosis model (Table 1). Biomaterial-based models have been developed, because they can be easily customized in 3D environment and engineered in various forms, such as films and tubular or porous structures, to meet the specific requirements of the study models. These models include the use of hydrogels and other polymers, where ECs and SMCs or PCs were cultivated together. A study of cocultured HUVECs and mesenchymal stem cells (MSCs) on the microperforated polycaprolactone (PCL) film revealed the up-regulation of α-smooth muscle actin (α-SMA) in the MSCs, suggesting the contribution of HUVECs in MSC differentiation during coculture [121]. Another example is the dual microstructured PCL film where both HUVEC and MSC were cocultured to examine the cell-to cell interaction through the porous channels in the PCL film. The result disclosed the increase of NO secretion [122]. Researchers also successfully cocultured HUVEC and adipose-derived stem cells (ADSCs) using thin SiO_2_ membrane (100 to 300 nm), which proved the potential as an atherosclerosis model [123]. Another model system harnessed elastic, porous, and ultrathin membrane (EPUM) where both HUVECs and human mesenchymal stem cells (hMSCs) were cocultured [124]. This study proved the functionality of endothelial barrier, as confirmed by the lower diffusion of fluorescein isothiocyanate (FITC)-dextran. These biomaterials have been employed mostly for assessing the robustness of coculture system. They lack more in-depth studies regarding the multicellular events during atherosclerosis development.

Apart from studying the functionality of ECs, biomaterial-based models are also useful in the simulation of leukocyte or monocyte transmigration through EC monolayer, which is a very early sign of atherosclerosis development. One such report is the use of nano- and microporous membrane to observe the transendothelial migration of leukocyte via the monolayer of ECs [125]. Another case is the use of porous polyethylene glycol diacrylate (PEGDA) hydrogel, where ECs responded to the TNF-α treatment with the up-regulation of ICAM-1 and VCAM-1[126]. This study also demonstrated transendothelial migration of leukocytes via ECs. Using 3D collagen matrix as the substrate, when researchers simulated the endothelial dysfunction using TNF-α and then seeded monocytes on the ECs, they observed the cell migration of monocytes and later noticed the differentiation into foam cells without the addition of lipids [87]. Biomaterial-based models have also been employed to replicate a series of the multicellular events, from endothelial dysfunction to early atherosclerosis formation. The use of in-bath coaxial cell printing enabled the coculture of ECs and SMCs [127]. Here, TNF-α-induced endothelial dysfunction and then subsequent addition of the monocytes proved transendothelial migration and their differentiation into macrophages. Moreover, upon the treatment of LDL, these macrophages turned to the foam cells as assessed by the accumulation of lipid uptake. In another study, researchers proposed an engineered endothelium model (EEM) where HUVECs and hMSCs were cocultivated on the porous membrane of polyvinyl alcohol (*p*-PVA) hydrogel embedded with decellularized ECM on both sides [128]. This study unveiled the cellular responses of ECs to the TNF-α treatment, as marked by the up-regulation of VCAM-1, ICAM, and loosening of VE-cad. After then, it also showed a resilient property of ECs with time. Upon the administration of TNF-α and Ox-LDL at specific time point, they also demonstrated the transendothelial migration of monocytes in their EEM and subsequent foam cell formation as well. In summary, biomaterial-based in vitro models are another promising tool for investigating the role of ECs in atherosclerosis progression. These models enable the mimicry of multicellular cascades, from endothelial dysfunction to foam cell formation.

### Animal models

For in vivo atherosclerosis study, genetically modified mice have been widely harnessed since the 1990s, notably with apolipoprotein E knockout (ApoE^−/−^) mice developed by Zhang et al. [129] and Plump et al. [130]. These models exhibit elevated cholesterol and spontaneous plaque formation on a high-fat diet [131–133]. Transgenic and conditional knockout (cKO) techniques further enable gene-specific manipulation of ECs, enhancing the physiological relevance for long-term monitoring of atherosclerosis progression [134–137]. Additionally, EC-specific gene manipulation has shed light on the study of endothelial dysfunction in atherosclerosis. For instance, overexpression of TFEB in ECs reduced VCAM-1 levels [138], while EC-PRAS40 knockout led to increased pro-inflammatory markers like VCAM-1 and ICAM-1 [105]. PECAM-1^−/−^ApoE^−/−^ mice disclosed reduced plaque formation and NF-κB activity under disturbed flow, indicating a pro-atherogenic role of PECAM-1 [139]. Similarly, excessive eNOS expression in ApoE^−/−^ eNOS-tg mice accelerated atherosclerosis due to heightened NO production [140]. The pro-inflammatory cytokine TNF-α would play a key role; TNF^−/−^ mice showed altered redox signaling that contributed to EC dysfunction [141], while Lepr^db^/db^TNF−^ mice confirmed TNF-α-induced ROS production and endothelial impairment [142]. Other nongenetic in vivo models, such as ApoE^−/−^ or C57BL/6J mice treated with agents like acetylcholine, Ca^2+^ ionophore, or diethylammonium (*Z*)-1-(*N*,*N*-diethylamino)diazen-1-ium-1,2-diolate (DEA-NONOate), simulate endothelial dysfunction through increased oxidative stress and reduced NO bioavailability [143]. Targeted gene modulation in ECs has also shown a therapeutic promise. BRCA1 overexpression in ECs via ad-BRCA1 declined plaque formation by limiting macrophage infiltration [144], while NEMO/IKKγ deletion decreased lesion development through NF-κB inhibition [145]. Nrf2’s dual roles were clarified via adeno-associated viral delivery in ApoE^−/−^ models, where its activation suppressed but its deletion promoted plaque progression [146]. Similarly, EC-specific deletion of TGF-β receptors [147] or HEG1 [148] suppressed plaque development, indicating their protective roles. Further, overexpression of Cav-1 led to lipid accumulation, inflammation, and reduced NO in ECs [149]. P2Y_2_R knockout promoted plaque stability [134], while TFPI-2 deficiency increased ECM degradation and inflammation via matrix metalloproteinase-1 (MMP-1) and MMP-9 up-regulation [135]. Taken together, in vivo model of atherosclerosis remains the best one over in vitro models, due to their capability to replicate the complex system and cellular interactions involved in atherosclerosis development (Table 2). Moreover, the in vivo model is by far the only one that can recapitulate advanced atherosclerosis stage. In addition, in vivo models remain indispensable for drug testing for atherosclerosis, because of their ability to manipulate EC-specific gene deletion or overexpression. Of course, there are some limitations of animal models, including species differences, time-consuming and costly process, inadequate equivalence to human, and ethical concerns [150].

## Therapeutic Approaches Targeting Endothelial Dysfunction

**Fig. 3.**
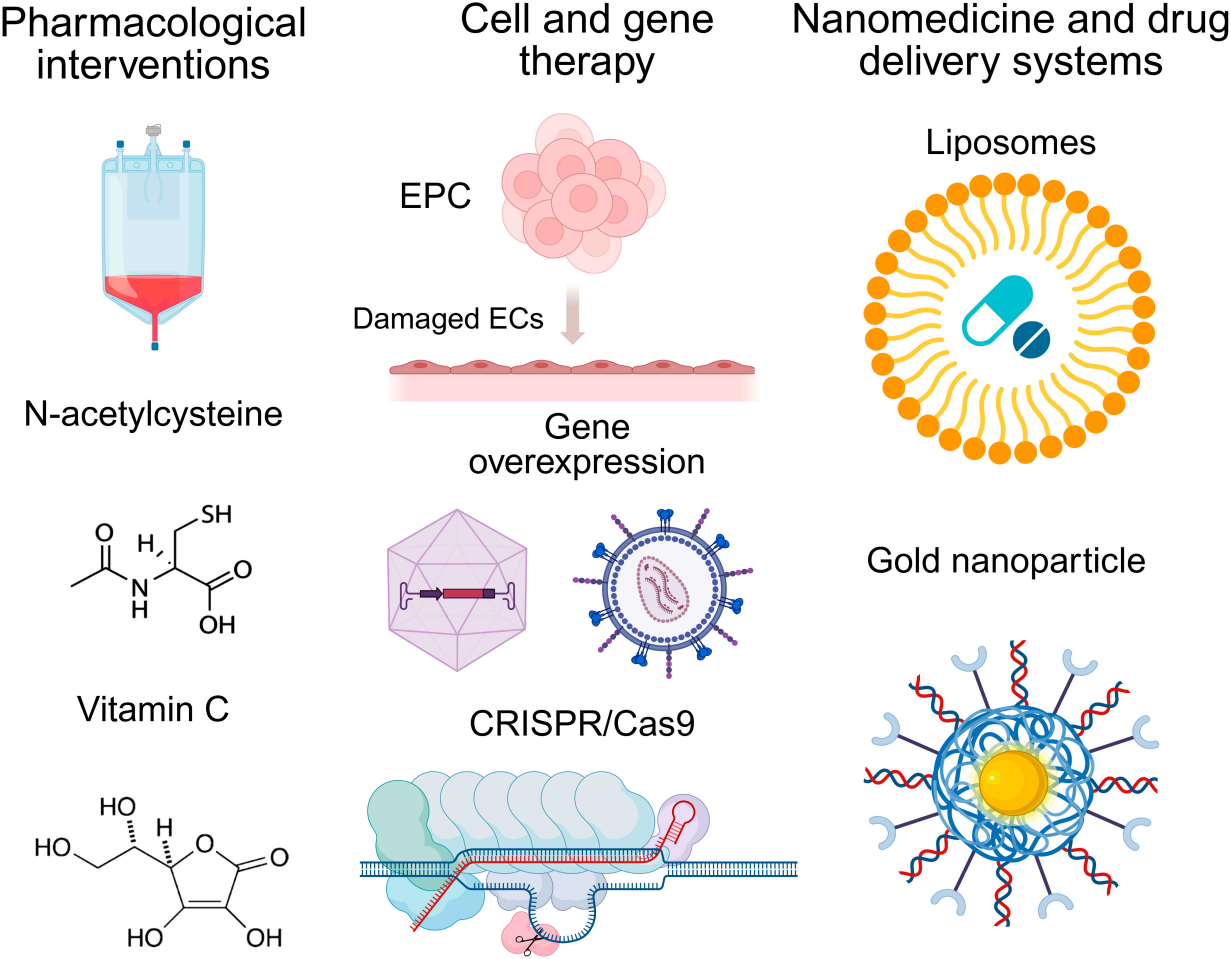
Examples of traditional and novel therapeutics, and intervention technologies for the treatment of atherosclerosis. A wide range of therapeutic strategies is being developed to restore endothelial function and mitigate atherosclerosis progression. Pharmacological interventions, such as N-acetylcysteine and vitamin C, help reduce oxidative stress, inflammation, and endothelial damage by scavenging ROS and enhancing NO bioavailability. Cell and gene therapy approaches focus on repairing damaged ECs and promoting vascular regeneration. EPC therapy supports endothelial repair, while specific gene overexpression and CRISPR/Cas9 gene editing aim key molecular pathways involved in endothelial dysfunction. Nanomedicine and advanced drug delivery systems, including liposomes and gold nanoparticles, enable precise and controlled drug release to atherosclerotic lesions, improving therapeutic efficacy while minimizing off-target effects. These innovative approaches hold a great promise for advancing vascular health and preventing cardiovascular disease progression. The image is produced using BioRender.

Endothelial dysfunction is a critical pathological development underlying a wide range of cardiovascular and metabolic disorders, including atherosclerosis, hypertension, diabetes, and chronic kidney disease. As such, understanding the complex interconnections between endothelial dysfunction and these diseases has been a focus of extensive research, leading to the development of various therapeutic approaches. This section address not only the conventional and cutting-edge therapeutics but also the intervention technologies for the treatment of atherosclerosis (Fig. 3).

### Pharmacological interventions

NO regulates leukocyte adhesion [151], platelet aggregation [152,153], and vasodilation, all of which are essential for vascular homeostasis. NO donors like NOS stimulators [154], sodium nitroprusside [155,156], and nitroglycerin [157] enhance endothelial function by increasing NO availability, reducing oxidative stress [158], inhibiting inflammation, and promoting vasodilation [159]. Oxidative stress reduces NO bioavailability by increasing ROS production [38,160], contributing significantly to endothelial dysfunction [161]. Antioxidants such as vitamin C [162,163], vitamin E [164], and N-acetylcysteine [165,166] counteract ROS and support NO-mediated pathways, improving endothelial function and reducing atherosclerosis progression and cardiovascular risk [167,168]. Statins, known for lowering cholesterol [169], also reduce inflammation [170] and improve endothelial function [171]. By blocking the mevalonate pathway [172], they down-regulate isoprenoid intermediates and up-regulate eNOS [173]. Statins enhance NO availability [174], reduce endothelial inflammation [170], and stabilize plaques, lowering the risk of thrombosis and cardiovascular events [175–177]. Anti-inflammatory therapies such as canakinumab [178] and colchicine [179] could suppress systemic inflammation, regardless of lipid levels. Combining lipid-lowering agents with targeted anti-inflammation offers additional endothelial protection. The renin–angiotensin–aldosterone system (RAAS) contributes to endothelial dysfunction via vasoconstriction [180], oxidative stress [181], and inflammation [182]. Angiotensin II receptor blockers (ARBs) [183] block RAAS activation, thus improving endothelial function by boosting NO [184,185], reducing oxidative stress [186,187], and suppressing cytokine release [188]. They also prevent adverse cardiac remodeling [189] and reduce cardiovascular symptoms [190]. Since inflammation is central to all stages of atherosclerosis, anti-inflammatory drugs such as corticosteroids [191], nonsteroidal anti-inflammatory medicines (NSAIDs) [192], and certain cytokine inhibitors [193] can suppress leukocyte migration [194], cytokine release, and endothelial activation. It should be noted that although those traditional pharmacological interventions are crucial for systemic atherosclerosis management, their efficacy is often hampered by off-target effects and poor bioavailability at the lesion site. As such, advances in biomaterial-based drug delivery systems, for example, biodegradable nanoparticles and drug-eluting stents, now enable localized and sustained release of these agents, significantly improving their therapeutic index within the atherosclerotic microenvironment [195]. These agents may serve as an adjunct therapy to stabilize plaques and prevent thrombosis.

### Cell and gene therapy

While targeting underlying genetic and cellular causes, cell and gene therapies have emerged as a viable therapeutic option for managing endothelial dysfunction in atherosclerosis. The basic concepts and therapeutic approaches in regaining endothelial function and retarding the progression of atherosclerosis are addressed in this section. For neovascularization and endothelialization of the damaged arteries, endothelial progenitor cells (EPCs) [196] are considered promising for endothelial repair and regeneration. Nevertheless, EPC function and quantity were vulnerable in atherosclerosis [197], resulting in poor endothelium repair and prevailing endothelial dysfunction. Exogenous EPC injection or mobilization is a key component of EPC-based therapy [198], which aims to improve endothelium regeneration and vascular repair. In preclinical and clinical trials, autologous EPC infusion or endogenous EPC mobilization as stimulated by G-CSF [199] showed a promise in encouraging endothelial repair and enhancing vascular function in atherosclerosis [200].

eNOS produces NO [201], which is a crucial regulator of vascular homeostasis. NO possesses vasodilatory [202], anti-inflammatory [203], and anti-thrombotic [204,205] properties. In atherosclerosis, decreased NO bioavailability and eNOS activity are crucial factors of endothelial dysfunction [206]. The goal of gene therapy is to increase eNOS expression and activity [207], which is intended to restore endothelial function and NO generation. In preclinical models, the delivery of eNOS-expressing vector, such as adenoviral or adeno-associated viral vector [208], to vascular ECs has proven effective in increasing vasodilation, lowering vascular inflammation [209], and minimizing the formation of atherosclerotic lesions. Targeting endothelial dysfunction in atherosclerosis is affordable using eNOS gene therapy, which is now undergoing clinical trials in patients with CVD [210].

The pathophysiology of atherosclerosis involves the dysregulation of several genes linked to endothelial dysfunction, including endothelin-1 (ET-1) [211], VCAM-1 [212], and ICAM-1 [213]. A promising option for modifying gene expression linked to endothelial dysfunction is antisense oligonucleotide (ASO) therapy [214–216]. By specifically inhibiting the expression of these genes, ASOs complementary to their mRNA sequences can reduce vascular inflammation, leukocyte adhesion, and endothelial activation. Preclinical research disclosed that ASO treatment was effective in the suppression of atherosclerotic lesions and enhancing vascular function [217,218]. The introduction of ASO-based therapy in clinical setting offers potential benefits for precision medicine in the treatment of atherosclerosis. Moreover, CRISPR-Cas9 and base editing, 2 recent developments in gene editing technology, also hold a great promise in repairing genetic flaws that underlie endothelial dysfunction and atherosclerosis risk. Targeted alteration of specific genes related to vascular inflammation [219], lipid metabolism [220], and endothelial function may present new therapeutic strategies for maintaining vascular homeostasis and delaying the development of atherosclerosis. Moreover, gene therapy techniques hold the potential to improve endothelial function, encourage vascular repair, and lessen the development of atherosclerotic lesions [221]. These techniques include the use of viral and nonviral vectors [222] to transfer therapeutic genes to ECs. The major goal of ongoing research is to ensure the safety, effectiveness, and specificity of gene editing and gene therapy technology for the management of cardiovascular illnesses linked to endothelial dysfunction.

It is notable that current cell and gene therapies hold immense promise for regenerating damaged endothelium and modulating the immune response in atherosclerosis. However, some challenges related to cell survival, engraftment, and targeted gene delivery are the major technical hurdles in ensuing successful clinical output of such treatments. Therefore, the role of biomaterials and carriers is crucial. Injectable hydrogels and biodegradable polymeric nanoparticles are the examples [223] that are designed to provide a supportive microenvironment for transplanted EPCs or to efficiently deliver therapeutic genes to the vascular wall, thus enhancing therapeutic outcomes.

### Nanomedicines and drug delivery systems

Beyond conventional pharmacological approaches, the emergence of nanomedicine has opened novel avenues for precise and targeted delivery of therapeutic agents in atherosclerosis (Table 3). These nanoparticle-based systems represent a significant intersection with biomaterials science, as their design involves the engineering of biocompatible and biodegradable materials to encapsulate, protect, and selectively deliver drugs or genes to atherosclerotic lesions, thereby minimizing off-target effects and enhancing therapeutic efficacy. These solutions provide distinct benefits in regard to overcoming biological barriers, enhancing medication absorption, or minimizing off-target effects by harnessing nanoscale materials and engineering principles. This section highlights representative nanomaterials and delivery platforms and describes how nanomedicine and drug delivery systems intersect to address endothelial dysfunction and progression of atherosclerosis, along with a brief description of imaging technologies.

**Table 3.** Summary of therapeutic approaches and their key actions/mechanisms

Therapeutics	Key action/mechanism	Refs.
NO donors	Increase NO levels, promoting vasodilation and reducing platelet aggregation	[154–157]
Antioxidants	Scavenge reactive oxygen species (ROS), improving NO bioavailability and endothelial function	[162–164]
Statins	Reduce inflammation and up-regulate endothelial nitric oxide synthase (eNOS), enhancing NO availability	[170,173,174]
ACE inhibitors/ARBs	Block the renin–angiotensin–aldosterone system (RAAS), leading to vasodilation and reduced oxidative stress	[186,187,189]
Anti-inflammatory drugs	Inhibit leukocyte migration and cytokine release, protecting endothelial function	[191–193]
Endothelial progenitor cells (EPCs)	Aid in neovascularization and repair of damaged endothelium	[198,199]
eNOS gene therapy	Increases expression of eNOS, enhancing NO production and improving vascular health	[206–208]
Antisense oligonucleotide (ASO) therapy	Reduces the expression of pro-inflammatory genes, decreasing inflammation	[214,215,218]
CRISPR and base editing	Targets and corrects genetic mutations associated with endothelial dysfunction	[221,222]
Lipid-based nanoparticles	Encapsulate hydrophobic drugs for effective delivery to atherosclerotic plaques	[227–229]
Polymeric nanoparticles	Offer flexible systems for delivering anti-inflammatory and gene therapies	[226,230]
Gold nanoparticles	Enable dual imaging and therapeutic delivery, enhancing targeting of endothelial cells	[235–237]
Nanotechnology	Allows for precise control over drug delivery and monitoring of vascular conditions	[239,240]

Liposomes [224], lipid nanoparticles [225], and lipid micelles [226] are the typical examples of lipid-based nanoparticles for the treatment of atherosclerosis. Hydrophobic medications, such as statins (e.g., simvastatin) [227], anti-inflammatory medicines (e.g., curcumin) [228], and antioxidants (e.g., resveratrol) [229], can be encapsulated in these nanoparticles to help them pass through endothelial barriers and accumulated in the atherosclerotic plaques. Lipid nanoparticles functionalized with targeting ligands are delivered to endothelial or inflammatory cells within the atherosclerotic lesions, improving therapeutic effectiveness and reducing systemic side effects.

Biocompatible polymers, poly-lactic-co-glycolic acid (PLGA) [230], and polyethylene glycol (PEG) [226] are also useful in fabricating polymeric nanoparticles, which enable flexible drug delivery for the treatment of atherosclerosis. A wide range of medicinal substances, including anti-inflammatory medications (e.g., dexamethasone) [231], anti-thrombotic medications (e.g., heparin) [232], and gene-based treatments (e.g., short interfering RNA) [233], can be encapsulated in these nanoparticles. By adding endothelium-targeted ligands to the surface of polymeric nanoparticles, their specificity for the ECs [234] that overexpress adhesion molecules in the atherosclerotic lesions was drastically improved, with better treatment prognosis and drug delivery efficiency.

Due to their optical and physicochemical characteristics, gold nanoparticles are another valuable option for drug administration and theragnostic purpose in atherosclerosis. By acting as carriers for therapeutic medications [235], imaging agents [236], and gene payloads [237], functionalized gold nanoparticles enable the simultaneous diagnosis and treatment of atherosclerotic lesions. The selective binding of gold nanoparticles to ECs or monocytes [238] within the atherosclerotic plaques is led by their conjugation with target ligands. Moreover, their stimuli-responsive features allow controlled drug release [239] and improve therapeutic efficacy [240] at target areas.

## Perspectives and Conclusions

Atherosclerosis involves complex multicellular events initiated by endothelial dysfunction. The role of ECs in the initiation, progression, and maturation of atherosclerosis has been extensively investigated using diverse experimental models, such as planar cell culture, microfluidics, OoC, biomaterial-based, and in vivo models. Despite these advancements, the development of novel experimental models is absolutely required in recapitulating the complex, multifactorial processes of atherosclerosis in a physiologically relevant manner.

Future direction of modeling should consider the integration of the strengths of various in vitro systems: microfluidics, OoC, and biomaterial-based model. Synergistic design and fabrication technology hold an immense potential to more accurately mimic multicellular interactions and the biomechanical environment of vascular tissues. Moreover, leveraging artificial intelligence and computational modeling may further substantially contribute to not only fabricating advanced models but also interpreting the data from these model systems and predicting disease outcomes. The therapeutic landscape for endothelial dysfunction, especially in the context of atherosclerosis, is expanding rapidly with a multifaceted approach combining pharmacological interventions, cell and gene therapies, nanomedicines, and emerging novel technologies. The future of endothelial dysfunction treatment lies in personalized and targeted therapies. Gene therapy, nanomedicine, and advanced drug delivery systems should enhance treatment precision, reduce side effects, and improve therapeutic efficacy.

Taken together, atherosclerosis is a cascade of multifactorial processes that involve various types of cells. We understand that modeling of such complex biological events is extremely challenging. To date, there are no perfect experimental models for the study of atherosclerosis. As such, it is recommendable that depending on the development stages of atherosclerosis, experimental models should be selectively employed to meet the specific goal of research and development. Sometimes, it may be wise to test 2 different models and cross-check the results to validate the outcomes. Collaborative efforts combining interdisciplinary expertise should be the key to the advanced therapeutics and innovative technologies in reducing the global burden of atherosclerosis.
